# FOLFOX Plus Bevacizumab Rechallenge Versus Regorafenib as Third-Line Treatment in Metastatic Colorectal Cancer: The Role of the Oxaliplatin-Free Interval

**DOI:** 10.3390/cancers18182987

**Published:** 2026-09-16

**Authors:** Ugur Ozkerim, Oguzcan Kınıkoglu, Sila Oksuz, Deniz Isik, Yunus Emre Altintas, Sedat Yildirim, Goncagul Akdag, Aslı Gecgel, Hatice Odabas, Tugba Basoglu, Nedim Turan

**Affiliations:** Department of Medical Oncology, Kartal Dr. Lütfi Kirdar City Hospital, University of Health Sciences, Istanbul 34865, Turkey; ogokinikoglu@yahoo.com (O.K.); sila.oksuz@gmail.com (S.O.); dnz.1984@yahoo.com (D.I.); yunusaltintas1688@gmail.com (Y.E.A.); rezansedat@hotmail.com (S.Y.); akdaggoncagul@gmail.com (G.A.); dr.asltrgt@gmail.com (A.G.); odabashatice@yahoo.com (H.O.); basoglutugba@gmail.com (T.B.); turan.nedim@hotmail.com (N.T.)

**Keywords:** metastatic colorectal cancer, oxaliplatin rechallenge, regorafenib, oxaliplatin-free interval, third-line treatment, progression-free survival

## Abstract

**Background:** The optimal use of chemotherapy rechallenge in later-line metastatic colorectal cancer (mCRC) remains uncertain. We compared third-line FOLFOX plus bevacizumab rechallenge with regorafenib and examined whether the oxaliplatin-free interval was associated with treatment outcomes. **Methods:** This retrospective single-center study included 122 patients with metastatic left-sided, RAS (KRAS/NRAS) and BRAF wild-type, proficient mismatch repair (pMMR) colorectal adenocarcinoma treated between 2018 and 2025. All patients had received first-line FOLFOX plus cetuximab or panitumumab, followed by FOLFIRI plus bevacizumab after progression. In the third-line setting, 65 patients received FOLFOX plus bevacizumab as oxaliplatin rechallenge and 57 received regorafenib. The primary endpoint was progression-free survival (PFS). Cox regression, propensity score-based inverse probability of treatment weighting (IPTW), and treatment-by-oxaliplatin-free interval interaction analyses were performed. **Results:** Median third-line PFS was 7.33 months with FOLFOX plus bevacizumab rechallenge and 6.17 months with regorafenib (log-rank *p* < 0.001). FOLFOX plus bevacizumab rechallenge was associated with a lower hazard of progression in multivariable analysis (adjusted HR, 0.58; 95% CI, 0.39–0.88; *p* = 0.009), with consistent results in the propensity score-based IPTW sensitivity analysis (HR, 0.58; 95% CI, 0.40–0.86; *p* = 0.006). Restricted cubic spline analysis indicated a nonlinear association between OFI and PFS (*p* for nonlinearity = 0.005), while flexible modeling did not provide robust evidence of a treatment-by-OFI interaction (global *p* = 0.806). In exploratory subgroup analyses, FOLFOX plus bevacizumab rechallenge was associated with longer PFS than regorafenib among patients with an OFI of ≥6 months (adjusted HR, 0.39; 95% CI, 0.24–0.65; *p* < 0.001), whereas no significant difference was observed among those with an OFI of <6 months (adjusted HR, 0.66; 95% CI, 0.35–1.25; *p* = 0.199). **Conclusions**: FOLFOX plus bevacizumab rechallenge was associated with longer third-line PFS than regorafenib in this selected and uniformly treated mCRC cohort. OFI was associated with PFS in a nonlinear manner, but there was no robust evidence that it modified the relative treatment effect across its continuous range. Exploratory subgroup findings based on a 6-month cutoff require external validation.

## Simple Summary

Treatment options become increasingly limited after standard first- and second-line therapies for metastatic colorectal cancer. Reusing oxaliplatin may be an option for selected patients, but it remains unclear which patients are most likely to benefit. In this study, we compared FOLFOX plus bevacizumab rechallenge with regorafenib as third-line treatment in 122 patients with left-sided, RAS wild-type metastatic colorectal cancer who had received a uniform sequence of previous therapies. Patients receiving FOLFOX plus bevacizumab rechallenge had longer progression-free survival than those receiving regorafenib. The oxaliplatin-free interval was also associated with progression-free survival, although this relationship was not linear. Exploratory analyses suggested different outcomes according to the duration of the oxaliplatin-free interval, but the data did not provide robust evidence that this interval specifically modified the relative treatment effect. Therefore, the oxaliplatin-free interval may provide useful prognostic and treatment-stratification information, but a specific cutoff should not currently be used to select treatment. Further studies are needed to validate these findings.

## 1. Introduction

Treatment options for metastatic colorectal cancer (mCRC) have expanded considerably over the past decade, particularly with the development of molecularly selected therapies. However, for patients without an actionable molecular target who have progressed after fluoropyrimidine-, oxaliplatin-, and irinotecan-based chemotherapy, the choice of later-line treatment remains challenging. Current guidelines include regorafenib and other later-line agents as standard options, while reintroducing a previously effective induction regimen may also be considered in selected patients [1,2].

Regorafenib was one of the first agents to demonstrate a survival benefit in refractory mCRC. In the phase III CORRECT trial, regorafenib improved overall survival compared with placebo in patients who had progressed after standard therapies [3]. The CONCUR trial subsequently confirmed this benefit [4]. Despite its established role, regorafenib’s clinical benefit is generally modest, and treatment-related adverse events may limit its use in some patients. These considerations have maintained interest in chemotherapy reintroduction as an alternative strategy in patients who previously benefited from cytotoxic treatment.

Oxaliplatin reintroduction is particularly relevant in this setting. Earlier studies suggested that sensitivity to oxaliplatin may be partly regained after a treatment-free interval. In the prospective RE-OPEN study, oxaliplatin reintroduction was feasible in patients who had previously achieved disease control with oxaliplatin-based therapy and had an oxaliplatin-free interval of at least 6 months [5]. A subsequent retrospective study of 110 patients also reported meaningful disease control with FOLFOX plus bevacizumab rechallenge in patients with an oxaliplatin-free interval of at least 6 months [6]. More recently, real-world studies have compared chemotherapy rechallenge with regorafenib or trifluridine/tipiracil, with some suggesting favorable outcomes in selected patients undergoing rechallenge [7,8]. Nevertheless, direct evidence specifically comparing FOLFOX plus bevacizumab rechallenge with regorafenib remains limited, and the clinical factors that may help identify patients most likely to benefit from oxaliplatin re-exposure are not well defined.

In this study, we compared progression-free survival with third-line FOLFOX plus bevacizumab rechallenge and regorafenib in patients with mCRC previously treated with oxaliplatin- and irinotecan-based chemotherapy. We also evaluated whether the oxaliplatin-free interval was associated with treatment outcomes and whether it could help identify patients in whom FOLFOX plus bevacizumab rechallenge may be a reasonable later-line treatment option.

## 2. Materials and Methods

### 2.1. Study Design and Patient Selection

This retrospective cohort study included patients with metastatic colorectal cancer treated at Kartal Dr. Lütfi Kırdar City Hospital between 2018 and 2025. We retrospectively reviewed the medical records of patients who received systemic treatment for metastatic disease during this period.

Eligibility criteria included histologically confirmed left-sided colorectal adenocarcinoma, RAS (KRAS/NRAS) and BRAF wild-type disease, proficient mismatch repair (pMMR) status, and receipt of a uniform treatment sequence during the first two lines of therapy. These molecular criteria were used to define a homogeneous population eligible for the same treatment strategy during the preceding lines of therapy and to minimize treatment-related heterogeneity before the third-line comparison. Testing for potentially actionable molecular alterations beyond RAS, BRAF, and MMR status was not performed systematically across the entire study population and was therefore not used as an eligibility criterion. All patients received FOLFOX combined with an anti-EGFR antibody (cetuximab or panitumumab) as first-line treatment, followed by FOLFIRI plus bevacizumab as second-line treatment. For this analysis, we included patients who subsequently received either FOLFOX plus bevacizumab rechallenge or regorafenib as third-line therapy.

Patients were required to have documented disease progression after second-line treatment and available clinical and radiological follow-up after initiation of third-line therapy. Patients with right-sided primary tumors, RAS- or BRAF-mutant disease, deficient mismatch repair (dMMR) status, non-adenocarcinoma histology, or third-line treatments other than FOLFOX plus bevacizumab rechallenge or regorafenib were excluded. Patients who were not considered suitable for FOLFOX plus bevacizumab rechallenge because of clinically significant residual oxaliplatin-related toxicity, particularly peripheral neuropathy, were also excluded. Thus, the analysis was restricted to patients who were considered clinically eligible for FOLFOX plus bevacizumab rechallenge at the time of third-line treatment initiation.

A total of 217 patients were screened. Of these, 95 were excluded: 4 because of a second primary malignancy, 11 because of non-cancer-related death before third-line treatment, and 80 because of progression during oxaliplatin-containing therapy or clinically significant oxaliplatin-related toxicity precluding rechallenge. Separate counts for progression and toxicity within the latter group were not available. The final cohort consisted of 122 patients, of whom 65 received FOLFOX plus bevacizumab rechallenge and 57 received regorafenib as third-line treatment.

### 2.2. Treatment and Clinical Variables

Demographic, disease-related, and treatment-related data were obtained from electronic medical records. Variables collected for this analysis included age, sex, ECOG performance status, metastatic sites at diagnosis, prior liver metastasectomy, first-line anti-EGFR agent, oxaliplatin-free interval, ECOG performance status before third-line treatment, and third-line treatment.

All patients received FOLFOX combined with cetuximab or panitumumab as first-line therapy. Oxaliplatin-containing treatment was administered for a planned duration of approximately 6 months and was discontinued after completion of the planned treatment period rather than because of disease progression. Patients then received maintenance treatment with 5-fluorouracil plus the same anti-EGFR agent (cetuximab or panitumumab) until disease progression. Following progression, all patients received FOLFIRI plus bevacizumab as second-line treatment. In patients without progression during combination therapy, FOLFIRI plus bevacizumab was continued for approximately 6 months, after which irinotecan was discontinued as planned and maintenance treatment with 5-fluorouracil plus bevacizumab was continued until disease progression.

After progression on second-line therapy, patients received either FOLFOX plus bevacizumab as oxaliplatin rechallenge or regorafenib as third-line treatment. Oxaliplatin rechallenge was defined as re-exposure to FOLFOX after an intervening oxaliplatin-free interval. Regorafenib was initiated at 80 mg once daily and escalated weekly, as tolerated, to a target dose of 160 mg once daily, administered on days 1–21 of each 28-day cycle. Dose modifications and treatment interruptions were made as clinically indicated.

The oxaliplatin-free interval was defined as the time between the last administration of oxaliplatin during first-line treatment and the initiation of third-line therapy. The interval was analyzed primarily as a continuous variable. A 6-month cutoff was additionally evaluated in an exploratory analysis based on previous studies of oxaliplatin reintroduction in metastatic colorectal cancer [5,6].

**Figure 1:**
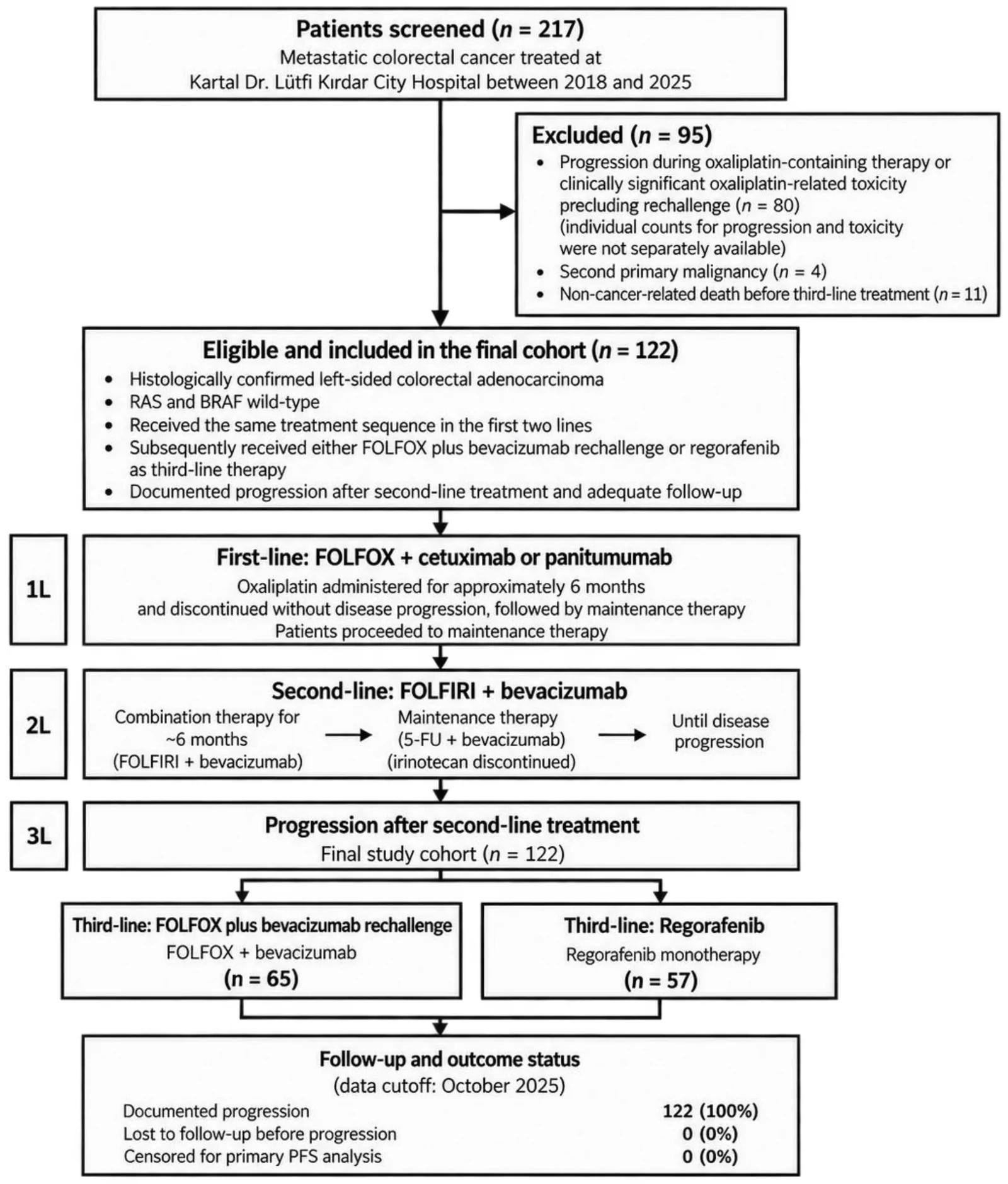
Study design and treatment sequence. The flow diagram summarizes the eligibility criteria, treatment sequence across the first three lines of therapy, and allocation of the final study cohort to FOLFOX plus bevacizumab rechallenge or regorafenib in the third-line setting. Abbreviations: 1L, first line; 2L, second line; 3L, third line; FOLFOX, folinic acid, fluorouracil, and oxaliplatin; FOLFIRI, folinic acid, fluorouracil, and irinotecan; 5-FU, 5-fluorouracil.

Figure 1 summarizes the study cohort and treatment sequence.

### 2.3. Outcome Definition

The primary outcome was third-line progression-free survival (3L-PFS), defined as the time from initiation of third-line treatment to documented disease progression or death from any cause, whichever occurred first. Tumor response and disease progression were assessed according to Response Evaluation Criteria in Solid Tumors (RECIST), version 1.1, based on routine radiological evaluations. Radiological assessments were routinely performed every 12 weeks during third-line treatment, with earlier imaging when clinically indicated because of symptoms or suspected disease progression. Disease progression was defined according to RECIST 1.1 as at least a 20% increase in the sum of diameters of target lesions relative to the smallest sum recorded during treatment, with an absolute increase of at least 5 mm, unequivocal progression of non-target lesions, or the appearance of one or more new lesions. The date of the first radiological assessment documenting progression was used as the progression date. All patients included in the study experienced documented disease progression, and no deaths occurred before progression; therefore, the primary PFS analysis had no censored observations. No otherwise-eligible patients were excluded because of the absence of a PFS event.

### 2.4. Statistical Analysis

Continuous variables were summarized as medians with interquartile ranges (IQRs) or means with standard deviations, as appropriate, while categorical variables were summarized as frequencies and percentages. Baseline characteristics were compared between the FOLFOX plus bevacizumab rechallenge and regorafenib groups using Student’s t-test or the Mann–Whitney U test for continuous variables, depending on data distribution. Categorical variables were compared using Fisher’s exact test for 2 × 2 contingency tables and the Fisher–Freeman–Halton exact test for variables with more than two categories. Standardized mean differences (SMDs) were calculated to assess baseline covariate balance between treatment groups, with absolute SMDs reported independently of hypothesis-testing *p* values.

Third-line PFS was estimated using the Kaplan–Meier method and compared between treatment groups using the log-rank test. Cox proportional hazards regression was used to estimate hazard ratios (HRs) with 95% confidence intervals (CIs). Univariable Cox regression analyses were first performed to examine the associations between individual clinical variables and third-line PFS. The proportional hazards assumption was assessed using Schoenfeld residuals.

A multivariable Cox regression model was used to evaluate the association between third-line treatment and PFS after adjustment for clinically relevant potential confounders. The model included treatment group, age, sex, ECOG performance status before third-line treatment, liver metastasis, peritoneal metastasis, and oxaliplatin-free interval.

The oxaliplatin-free interval was modeled primarily as a continuous variable. Its functional form was further evaluated using restricted cubic splines with three knots at 5, 6, and 7 months. A likelihood-ratio test comparing the spline model with the corresponding linear model was used to assess evidence of nonlinearity. As an additional sensitivity analysis, the principal multivariable Cox model was refitted with OFI modeled using restricted cubic splines with knots at 5, 6, and 7 months rather than as a linear covariate. To evaluate whether the relative association between third-line treatment and PFS varied across the continuous oxaliplatin-free interval, treatment-by-OFI interaction models were fitted using the same covariate set as the principal multivariable model. The interaction was evaluated using both a linear treatment-by-OFI term and, as a flexible analysis, treatment-by-restricted-cubic-spline terms. Model-based treatment hazard ratios with 95% confidence intervals were estimated across the observed OFI range. In an additional exploratory analysis, patients were stratified according to an oxaliplatin-free interval of <6 months versus ≥6 months. Given the limited subgroup sample sizes, subgroup-specific Cox models were adjusted for age, sex, and ECOG performance status before third-line treatment to provide a parsimonious adjustment.

As a sensitivity analysis, inverse probability of treatment weighting (IPTW) based on the propensity score was performed. The propensity score was estimated using a logistic regression model including age, sex, ECOG performance status before third-line treatment, liver metastasis, peritoneal metastasis, and oxaliplatin-free interval. Stabilized inverse probability weights were calculated and used in a weighted Cox proportional hazards model to estimate the association between third-line treatment and PFS. Propensity score distributions and common support between treatment groups were examined to assess overlap and identify extreme propensity scores. Covariate balance before and after weighting was evaluated using absolute standardized mean differences (SMDs), with an absolute SMD <0.10 considered indicative of adequate balance.

There were no missing data for any variables included in the analyses. Therefore, no imputation was performed, and all descriptive, multivariable Cox regression, and propensity score-based IPTW analyses included the complete cohort of 122 patients.

All statistical tests were two-sided, and *p* < 0.05 was considered statistically significant. Statistical analyses were performed using Python (version 3.13.5).

### 2.5. Ethical Considerations

This study was conducted in accordance with the Declaration of Helsinki and was approved by the Ethics Committee of Kartal Dr. Lütfi Kırdar City Hospital (Decision No. 2025/010.99/15/11; 30 April 2025). The requirement for informed consent was waived by the Ethics Committee due to the retrospective nature of the study.

## 3. Results

### 3.1. Patient Characteristics

A total of 122 patients met the eligibility criteria and were included in the analysis. Of these, 65 patients (53.3%) received FOLFOX plus bevacizumab as oxaliplatin rechallenge, whereas 57 (46.7%) received regorafenib as third-line treatment. At the data cutoff in October 2025, all 122 patients had experienced documented disease progression. No patients were lost to follow-up before progression, and there were no censored observations in the primary PFS analysis.

The median age was 57 years (IQR, 46–66) in the FOLFOX plus bevacizumab rechallenge group and 60 years (IQR, 49–67) in the regorafenib group (*p* = 0.224). Men accounted for 55.4% and 56.1% of the two groups, respectively. Baseline ECOG performance status was also similar between groups, with most patients having an ECOG performance status of 0 or 1.

At diagnosis, liver metastases were present in 73.8% of patients in the FOLFOX plus bevacizumab rechallenge group and in 78.9% in the regorafenib group. The corresponding rates of lung metastases were 16.9% and 24.6%, and those of peritoneal metastases were 35.4% and 28.1%, respectively. Previous liver metastasectomy had been performed in 23.1% and 26.3% of patients, respectively. The first-line anti-EGFR agent was similarly distributed between the treatment groups.

The median oxaliplatin-free interval was 6 months in both groups, although its distribution tended to be longer in patients who subsequently received FOLFOX plus bevacizumab rechallenge (IQR, 5–7 months) than in those who received regorafenib (IQR, 5–6 months; *p* = 0.056). ECOG performance status immediately before third-line treatment was comparable between groups; 60 of 65 patients (92.3%) in the rechallenge group and 53 of 57 (93.0%) in the regorafenib group had an ECOG performance status of 1.

Baseline covariate balance was additionally assessed using standardized mean differences (SMDs). Although several characteristics showed relatively small differences between groups, larger imbalances were observed for age, liver metastasis, lung metastasis, peritoneal metastasis, and particularly the oxaliplatin-free interval (Table 1).

### 3.2. Third-Line Progression-Free Survival

During third-line treatment, all 122 patients progressed. Median PFS was 7.33 months (95% CI, 6.90–9.43) in the FOLFOX plus bevacizumab rechallenge group and 6.17 months (95% CI, 5.50–7.10) in the regorafenib group. Kaplan–Meier analysis showed longer PFS with FOLFOX plus bevacizumab rechallenge than with regorafenib (log-rank *p* < 0.001).

**Figure 2:**
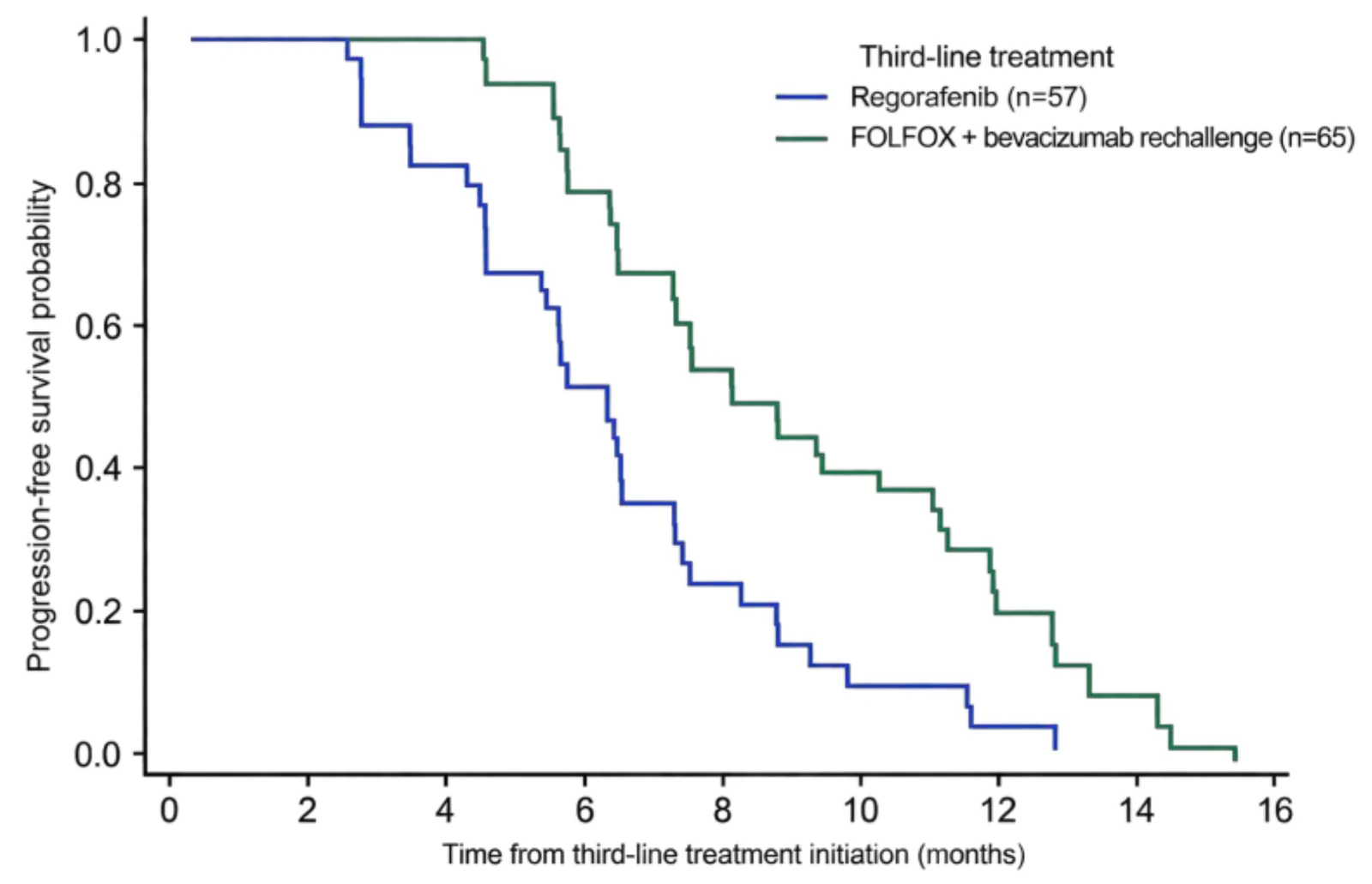
Kaplan–Meier analysis of progression-free survival according to third-line treatment. Median progression-free survival was 7.33 months (95% CI, 6.90–9.43) in the FOLFOX plus bevacizumab rechallenge group and 6.17 months (95% CI, 5.50–7.10) in the regorafenib group (log-rank *p* < 0.001). Abbreviations: PFS, progression-free survival.

In univariable Cox regression analysis, FOLFOX plus bevacizumab rechallenge was associated with a lower hazard of disease progression compared with regorafenib (HR, 0.45; 95% CI, 0.30–0.67; *p* < 0.001). Figure 2 shows the Kaplan–Meier curves for third-line PFS by treatment group.

### 3.3. Multivariable Analysis

In multivariable Cox regression analysis, FOLFOX plus bevacizumab rechallenge remained associated with a lower hazard of disease progression compared with regorafenib after adjustment for age, sex, ECOG performance status before third-line treatment, liver metastasis, peritoneal metastasis, and oxaliplatin-free interval (adjusted HR, 0.58; 95% CI, 0.39–0.88; *p* = 0.009). We observed no evidence of violation of the proportional hazards assumption for the treatment variable, and the global proportional hazards test was non-significant.

The oxaliplatin-free interval had a median of 6 months (IQR, 5–6 months) and ranged from 4 to 9 months. When modeled linearly, a longer oxaliplatin-free interval was associated with a lower hazard of progression. However, restricted cubic spline analysis provided evidence of nonlinearity in the association between oxaliplatin-free interval and PFS (*p* for nonlinearity = 0.005), indicating that a constant hazard ratio per 1-month increase did not adequately describe the relationship across the observed range.

Age, sex, ECOG performance status before third-line treatment, liver metastasis, and peritoneal metastasis were not significantly associated with third-line PFS in the multivariable model. Table 2 summarizes the univariable and multivariable Cox regression results.

**Figure 3:**
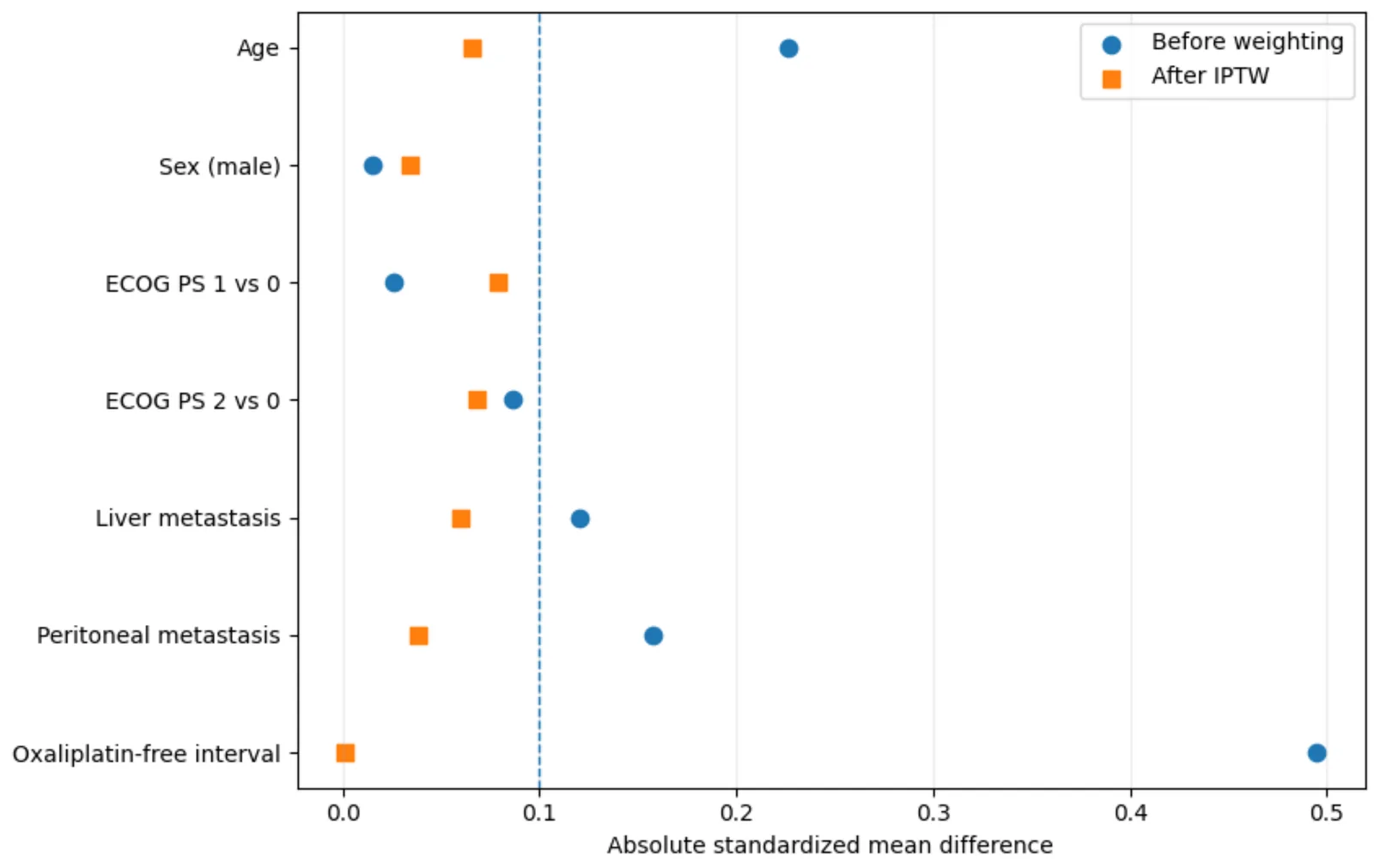
Covariate balance before and after inverse probability of treatment weighting (IPTW). Absolute standardized mean differences (SMDs) are shown for covariates included in the propensity score model. The dashed vertical line indicates an absolute SMD of 0.10. All evaluated covariates had an absolute SMD <0.10 after weighting.

In the propensity score-based IPTW sensitivity analysis, propensity score distributions showed overall overlap between the treatment groups, although overlap was reduced at the extremes. The empirical common support range was 0.321–0.887 (Supplementary Figure S1). Only 4 of 122 patients were outside the common support range, and extreme propensity scores were uncommon. Stabilized weights ranged from 0.588 to 4.137, with an effective sample size of 107.4. After weighting, all covariates included in the propensity score model achieved an absolute SMD < 0.10. (Figure 3). The association between FOLFOX plus bevacizumab rechallenge and improved PFS remained consistent in the IPTW-weighted Cox analysis (HR, 0.58; 95% CI, 0.40–0.86; *p* = 0.006).

### 3.4. Oxaliplatin-Free Interval and Treatment Association

Exploratory analyses were performed to assess whether the association between third-line treatment and PFS varied according to the duration of the oxaliplatin-free interval. In the linear interaction model using the same covariate set as the principal multivariable model, the treatment-by-oxaliplatin-free interval interaction coefficient was −0.310, corresponding to a multiplicative HR of 0.73 per 1-month increase in OFI (95% CI, 0.53–1.01; *p* = 0.058). When the relationship was modeled flexibly using restricted cubic splines, there was no evidence of an overall treatment-by-OFI interaction (global *p* = 0.806). Thus, the data did not provide robust evidence that the relative treatment effect varied continuously across the observed OFI range. In a sensitivity analysis, the association between FOLFOX plus bevacizumab rechallenge and longer PFS remained materially unchanged when OFI was modeled using restricted cubic splines rather than as a linear covariate (adjusted HR, 0.56; 95% CI, 0.37–0.83; *p* = 0.004) (Table 3).

Using the exploratory cutoff of 6 months, 40 patients had an oxaliplatin-free interval of <6 months, including 20 patients in each treatment group. In this subgroup, median PFS was 7.12 months with FOLFOX plus bevacizumab rechallenge and 5.80 months with regorafenib. The difference was not statistically significant in either the unadjusted analysis (HR, 0.66; 95% CI, 0.35–1.24; *p* = 0.199) or the adjusted analysis (adjusted HR, 0.66; 95% CI, 0.35–1.25; *p* = 0.199).

**Figure 4:**
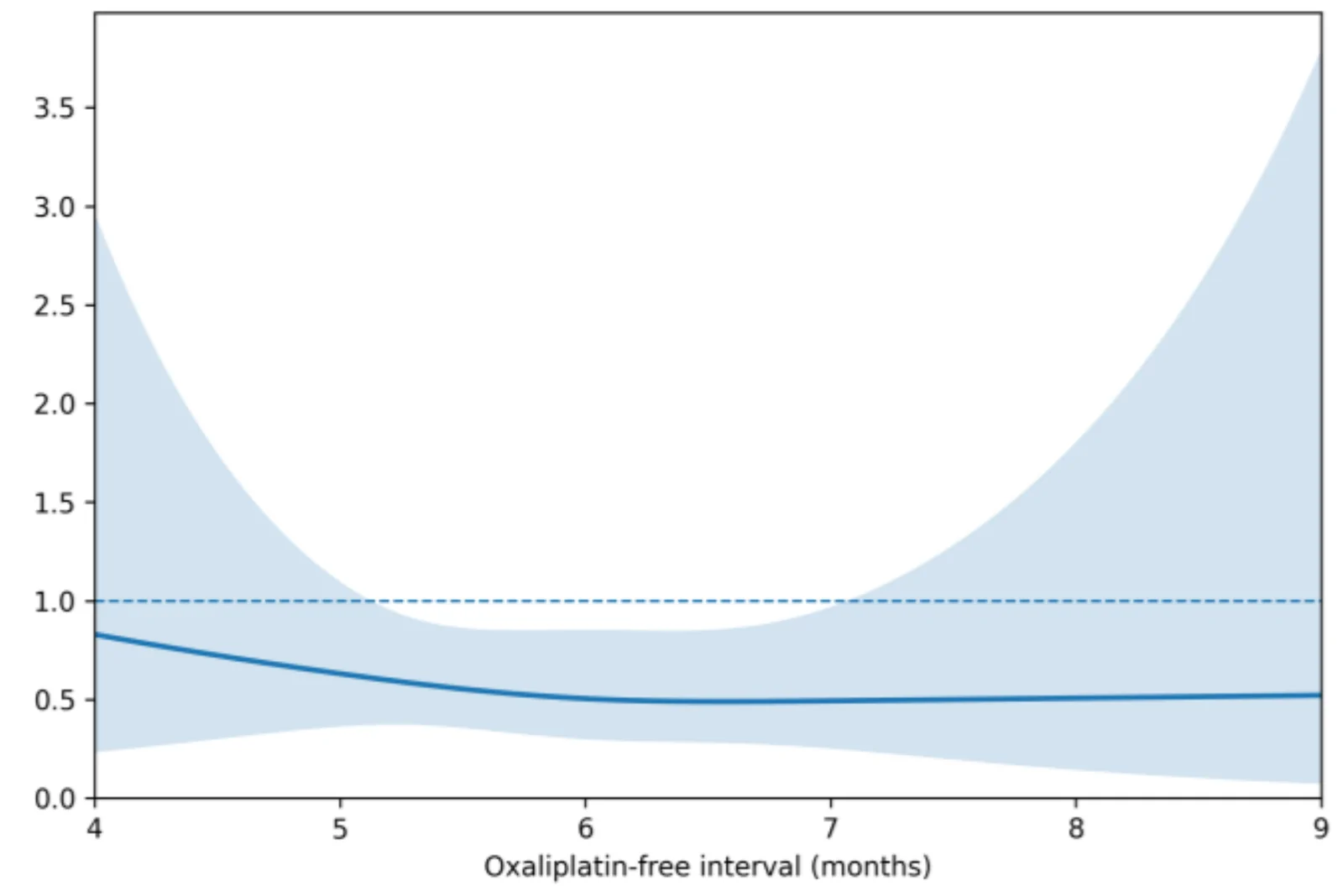
Modeled treatment effect across the observed continuous oxaliplatin-free interval. Adjusted hazard ratios (HRs) for progression-free survival comparing FOLFOX plus bevacizumab rechallenge with regorafenib were estimated using a restricted cubic spline interaction model. The solid line represents the adjusted HR, and the shaded area represents the 95% confidence interval. The dashed horizontal line indicates HR = 1.0. OFI, oxaliplatin-free interval.

Among the 82 patients with an oxaliplatin-free interval of ≥6 months, 45 received FOLFOX plus bevacizumab and 37 received regorafenib. Median PFS was 7.80 months and 6.20 months, respectively. In this subgroup, FOLFOX plus bevacizumab rechallenge was associated with a lower hazard of progression in the unadjusted analysis (HR, 0.40; 95% CI, 0.24–0.65; *p* < 0.001), and the association remained after adjustment for age, sex, and ECOG performance status before third-line treatment (adjusted HR, 0.39; 95% CI, 0.24–0.65; *p* < 0.001) (Figure 4).

## 4. Discussion

In this retrospective cohort of patients with left-sided, RAS wild-type metastatic colorectal cancer who had received a uniform treatment sequence during the first two lines of therapy, FOLFOX plus bevacizumab rechallenge was associated with longer third-line PFS than regorafenib. This association remained significant after adjustment for relevant clinical characteristics and was consistent in the propensity score-based IPTW sensitivity analysis. The oxaliplatin-free interval was associated with PFS, although restricted cubic spline analysis indicated that this relationship was nonlinear. Furthermore, flexible modeling did not provide robust evidence of treatment-effect modification across the continuous oxaliplatin-free interval. In the exploratory analysis using a 6-month cutoff, the association favoring rechallenge was observed in patients with an oxaliplatin-free interval of ≥6 months, whereas no statistically significant difference was observed in those with a shorter interval. These subgroup findings should therefore be considered exploratory rather than evidence of an established treatment-selection threshold. Because the rechallenge regimen consisted of FOLFOX plus bevacizumab, the observed treatment association should be interpreted as reflecting the combined regimen and cannot be attributed specifically to oxaliplatin alone.

These findings are consistent with previous evidence showing that re-exposure to oxaliplatin can retain clinical activity in selected patients with mCRC. The prospective RE-OPEN study demonstrated the feasibility of oxaliplatin reintroduction after an oxaliplatin-free interval of at least 6 months [5], while Kim et al. reported an objective response rate of 30.9% and a disease control rate of 68.2% in 110 patients undergoing oxaliplatin rechallenge [6]. A systematic review subsequently confirmed the potential activity of oxaliplatin retreatment, although outcomes varied considerably across studies, highlighting the importance of patient selection [9]. More recently, the RETROX-CRC study reported a response rate of 21.6%, disease control rate of 57.8%, and median PFS of 5.1 months with oxaliplatin retreatment [10]. Additional real-world evidence has also demonstrated clinically meaningful disease control with oxaliplatin re-exposure in later treatment lines, although outcomes vary according to patient selection and prior treatment history [11,12].

Comparative real-world evidence is also emerging. The PROSERpYNa study found longer PFS and OS with chemotherapy rechallenge or reintroduction than with regorafenib or trifluridine/tipiracil, and this association persisted after propensity score adjustment [13]. Other multicenter retrospective studies have similarly evaluated chemotherapy rechallenge against established later-line agents, although differences in treatment regimens, prior treatment exposure, and patient selection complicate direct comparisons [7,8,14]. Our study adds to this evidence by focusing on a more homogeneous population with a uniform first- and second-line treatment sequence and by specifically examining oxaliplatin-free interval as a potential treatment-selection factor.

The oxaliplatin-free interval may be particularly relevant in this context. In a pooled analysis of the OPTIMOX1 and OPTIMOX2 studies, Chibaudel et al. reported higher response rates and longer PFS after oxaliplatin reintroduction in patients with an oxaliplatin-free interval of ≥6 months than in those with a shorter interval [15]. This observation provided a clinical basis for considering the treatment-free interval when selecting patients for oxaliplatin re-exposure. In our study, restricted cubic spline analysis indicated a nonlinear association between the oxaliplatin-free interval and PFS. Although the linear treatment-by-OFI interaction suggested a possible trend toward greater relative benefit with increasing OFI (interaction HR, 0.73; 95% CI, 0.53–1.01; *p* = 0.058), the flexible spline interaction was not significant (global *p* = 0.806). In exploratory subgroup analyses, FOLFOX plus bevacizumab rechallenge was associated with a lower adjusted hazard of progression among patients with an OFI of ≥6 months (adjusted HR, 0.39), whereas no statistically significant difference was observed among those with an OFI of <6 months. These findings do not establish 6 months as a definitive treatment-selection threshold and should be considered hypothesis-generating. Nevertheless, the nonlinear association between OFI and PFS supports further investigation of OFI as a clinically accessible prognostic and potential treatment-stratification factor.

The treatment history of our cohort is also important when interpreting these findings. Oxaliplatin was discontinued after completion of the planned first-line treatment period rather than because of disease progression, followed by maintenance therapy. Thus, these patients were not selected for re-exposure after documented progression on oxaliplatin. This treatment pattern is consistent with the broader stop-and-go concept established in mCRC. In OPTIMOX1, planned interruption of oxaliplatin with subsequent reintroduction maintained treatment efficacy while reducing continuous exposure [16], and subsequent analyses suggested an association between oxaliplatin reintroduction and improved survival [17]. The feasibility of a planned oxaliplatin stop-and-go strategy followed by maintenance therapy and subsequent oxaliplatin reintroduction has also been demonstrated in a prospective phase II study [18]. The oxaliplatin-free interval in our cohort may therefore reflect not only time without drug exposure but also prior treatment sensitivity and underlying disease biology. Importantly, because OFI encompassed maintenance therapy, second-line treatment, and subsequent disease progression before third-line therapy, a longer interval may partly represent a more favorable disease course rather than restoration of oxaliplatin sensitivity specifically. Accordingly, OFI should primarily be regarded as a prognostic and treatment-stratification factor in the present study, rather than as an established predictive biomarker of benefit from FOLFOX plus bevacizumab rechallenge.

The present results should not be interpreted as evidence that FOLFOX plus bevacizumab rechallenge should routinely replace approved later-line therapies. Regorafenib has demonstrated a survival benefit in randomized trials [3,4], and subsequent large prospective studies have confirmed its activity and provided additional safety information in refractory mCRC [19,20]. Dose-optimization strategies have also been investigated to improve regorafenib tolerability without compromising activity [21]. Meanwhile, the later-line treatment landscape has expanded substantially. In the SUNLIGHT trial, trifluridine/tipiracil plus bevacizumab improved median OS from 7.5 to 10.8 months and median PFS from 2.4 to 5.6 months compared with trifluridine/tipiracil alone [22]. The efficacy of trifluridine/tipiracil monotherapy had previously been established in the phase III RECOURSE trial [23], and randomized phase II data had already suggested additional benefit from combining trifluridine/tipiracil with bevacizumab [24]. Fruquintinib also improved survival in heavily pretreated patients in the phase III FRESCO-2 trial [25,26], consistent with the earlier phase III FRESCO study [27]. Real-world studies have further emphasized that the optimal sequencing of available later-line agents remains uncertain and should be individualized according to patient and disease characteristics [28]. A large US real-world analysis likewise found no significant difference in survival between regorafenib and trifluridine/tipiracil after propensity score weighting, further supporting individualized treatment selection [29]. Chemotherapy rechallenge should therefore be considered as one component of an increasingly individualized treatment continuum rather than as a universal alternative to these agents [30]. This approach is also consistent with expert consensus emphasizing treatment sequencing, preservation of quality of life, and the ability to maintain subsequent treatment options in third-line mCRC [31].

An important strength of our study is the homogeneity of the study population. All patients had left-sided, RAS wild-type adenocarcinoma and received FOLFOX plus an anti-EGFR antibody followed by FOLFIRI plus bevacizumab before third-line treatment. This reduces some of the treatment and molecular heterogeneity encountered in previous retrospective rechallenge series. In addition, the treatment association remained consistent in both the multivariable analysis and the propensity score-based IPTW sensitivity analysis. However, propensity score methods can account only for measured covariates and cannot eliminate confounding by indication arising from unmeasured factors. Treatment selection may have been influenced by clinically relevant factors not fully captured in the dataset, including the depth and duration of response to prior therapy, residual oxaliplatin-related toxicity, comorbidities, physician judgment, and patient preference. Therefore, residual unmeasured confounding remains an important limitation of this nonrandomized comparison.

## 5. Limitations

This study has several limitations. Its retrospective, single-center design and relatively modest sample size limit causal inference and external generalizability. Treatment allocation was not randomized, and residual confounding, including confounding by indication, may remain despite multivariable adjustment and propensity score-based IPTW. In addition, the oxaliplatin-free interval may partly reflect the underlying disease tempo and the duration of benefit from preceding therapies, rather than oxaliplatin sensitivity alone. Detailed measures of prior treatment sensitivity, including the depth and duration of response to first-line therapy, duration of maintenance therapy, and second-line PFS, were not systematically available in the dataset and therefore could not be incorporated into the multivariable analyses. This limits our ability to distinguish whether OFI represents a treatment-specific marker of benefit from rechallenge or a more general marker of favorable disease biology. The analysis using a 6-month oxaliplatin-free interval cutoff was exploratory, and flexible modeling did not demonstrate robust treatment-effect modification across the continuous OFI range. Therefore, the subgroup findings should not be interpreted as establishing a definitive cutoff for treatment selection.

Detailed safety data during third-line treatment, including peripheral neuropathy, dose reductions, relative dose intensity, cumulative oxaliplatin exposure, grade ≥3 adverse events, and treatment discontinuations due to toxicity, were not systematically available and therefore could not be compared between treatment groups. Although patients with clinically significant residual oxaliplatin-related toxicity precluding rechallenge were excluded, the absence of detailed on-treatment safety and treatment-exposure data limits a comprehensive assessment of the risk–benefit balance between the two strategies and may also contribute to residual treatment-selection bias.

Radiological assessments were routinely performed every 12 weeks, with earlier imaging when clinically indicated because of symptoms or suspected progression. However, actual patient-level imaging intervals were not systematically available for comparison between treatment groups. Therefore, differential surveillance intensity cannot be excluded, and potential assessment-time bias may have influenced the measured PFS.

Finally, the study focused on PFS and did not evaluate OS as a comparative endpoint, and subsequent therapies could influence longer-term outcomes.

Despite these limitations, the findings support further evaluation of FOLFOX plus bevacizumab rechallenge as a third-line option in appropriately selected patients who previously discontinued oxaliplatin without progression. The oxaliplatin-free interval may provide clinically relevant prognostic and treatment-stratification information, but its role and the relevance of a 6-month threshold require external validation in larger multicenter cohorts.

## 6. Conclusions

In this retrospective cohort, FOLFOX plus bevacizumab rechallenge was associated with longer third-line PFS than regorafenib in patients with left-sided, RAS wild-type metastatic colorectal cancer who had previously discontinued oxaliplatin without progression. The oxaliplatin-free interval was associated with PFS in a nonlinear manner, but flexible modeling did not provide robust evidence that OFI modified the relative treatment effect across its continuous range. In exploratory subgroup analyses, the association favoring FOLFOX plus bevacizumab rechallenge was observed in patients with an OFI of ≥6 months, whereas no statistically significant difference was observed in those with a shorter interval. These hypothesis-generating findings support further evaluation of FOLFOX plus bevacizumab rechallenge in appropriately selected patients and of OFI as a clinically accessible prognostic and treatment-stratification factor. Prospective or multicenter validation is required before a specific OFI threshold can be used to guide treatment selection.

## Figures and Tables

**Table 1:** Baseline clinical and treatment characteristics according to third-line treatment.

Characteristic	FOLFOX Plus Bevacizumab Rechallenge (*n* = 65)	Regorafenib (*n* = 57)	*p* Value	SMD
Age, years, median (IQR)	57 (46–66)	60 (49–67)	0.224	0.226
Sex, *n* (%)			1.000	0.015
Male	36 (55.4)	32 (56.1)		
Female	29 (44.6)	25 (43.9)		
Baseline ECOG PS, *n* (%)			1.000	0.032
0	34 (52.3)	29 (50.9)		
1	30 (46.2)	27 (47.4)		
2	1 (1.5)	1 (1.8)		
Liver metastasis at diagnosis, *n* (%)	48 (73.8)	45 (78.9)	0.531	0.120
Lung metastasis at diagnosis, *n* (%)	11 (16.9)	14 (24.6)	0.370	0.189
Peritoneal metastasis at diagnosis, *n* (%)	23 (35.4)	16 (28.1)	0.440	0.158
Previous liver metastasectomy, *n* (%)	15 (23.1)	15 (26.3)	0.833	0.075
First-line anti-EGFR agent, *n* (%)			1.000	0.011
Cetuximab	38 (58.5)	33 (57.9)		
Panitumumab	27 (41.5)	24 (42.1)		
Oxaliplatin-free interval, months, median (IQR)	6 (5–7)	6 (5–6)	0.056	0.495
ECOG PS before third-line treatment, *n* (%)			1.000	0.090
0	3 (4.6)	3 (5.3)		
1	60 (92.3)	53 (93.0)		
2	2 (3.1)	1 (1.8)		

Abbreviations: ECOG PS, Eastern Cooperative Oncology Group performance status; IQR, interquartile range; SMD, standardized mean difference. SMDs are presented as absolute values.

**Table 2:** Univariable and multivariable Cox regression analyses for third-line progression-free survival.

Variable	Univariable HR (95% CI)	*p* Value	Multivariable HR (95% CI)	*p* Value
Third-line treatment: FOLFOX plus bevacizumab vs. regorafenib	0.45 (0.30–0.67)	<0.001	0.58 (0.39–0.88)	0.009
Age, per year	1.01 (0.99–1.02)	0.218	1.00 (0.99–1.02)	0.638
Male vs. female	0.95 (0.66–1.37)	0.802	0.95 (0.63–1.42)	0.795
ECOG PS before third-line treatment, per 1-point increase	1.07 (0.46–2.47)	0.883	1.04 (0.42–2.58)	0.937
Liver metastasis at diagnosis, yes vs. no	1.42 (0.93–2.18)	0.104	1.29 (0.79–2.12)	0.315
Peritoneal metastasis at diagnosis, yes vs. no	0.73 (0.50–1.07)	0.111	0.85 (0.57–1.29)	0.453

Abbreviations: CI, confidence interval; ECOG PS, Eastern Cooperative Oncology Group performance status; HR, hazard ratio.

**Table 3:** Third-line progression-free survival according to oxaliplatin-free interval subgroup.

OFI Subgroup	Sample Size	Unadjusted HR (95% CI)	*p* Value	Adjusted HR (95% CI)	*p* Value
<6 months	*n* = 40 (FOLFOX plus bevacizumab rechallenge: 20; regorafenib: 20)	0.66 (0.35–1.24)	0.199	0.66 (0.35–1.25)	0.199
≥6 months	*n* = 82 (FOLFOX plus bevacizumab rechallenge: 45; regorafenib: 37)	0.40 (0.24–0.65)	<0.001	0.39 (0.24–0.65)	<0.001

Linear treatment-by-continuous OFI interaction: β = −0.310; multiplicative HR, 0.73 (95% CI, 0.53–1.01); *p* = 0.058. Flexible treatment-by-OFI interaction using restricted cubic splines: global *p* = 0.806. Abbreviations: CI, confidence interval; HR, hazard ratio; OFI, oxaliplatin-free interval.

## Data Availability

The data presented in this study are available on request from the corresponding author.

## Supplementary Materials

The following supporting information can be downloaded at: https://www.mdpi.com/article/10.3390/cancers18182987/s1, Figure S1: Distribution and overlap of estimated propensity scores according to third-line treatment group. Dashed vertical lines indicate the empirical boundaries of the common support region (0.321–0.887), defined by the overlapping range of propensity scores observed in the two treatment groups. No patients were excluded on the basis of these boundaries.
