# Supplementary material for: FOLFOX Plus Bevacizumab Rechallenge Versus Regorafenib as Third-Line Treatment in Metastatic Colorectal Cancer: The Role of the Oxaliplatin-Free Interval
Source: Cancers (Basel). 2026 Sep 16;18(18):2987. doi: 10.3390/cancers18182987 (PMC13604701; doi:10.3390/cancers18182987)
Supplement: Supplementary file 1 [file cancers-18-02987-s001.zip › cancers-4555073-supplementary.pdf]

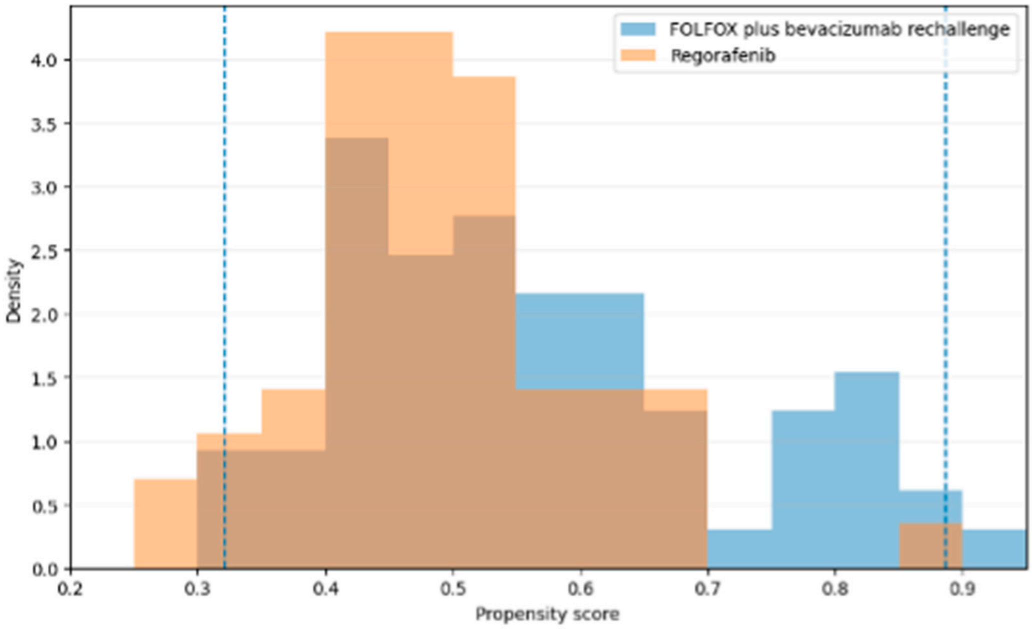

Supplementary Figure S1. Distribution and overlap of estimated propensity scores according to third-line treatment group. Dashed vertical lines indicate the empirical boundaries of the common support region (0.321–0.887), defined by the overlapping range of propensity scores observed in the two treatment groups. No patients were excluded on the basis of these boundaries.
